# Temperature‐dependent extraction kinetics of hydrolyzed collagen from scales of croaker fish using thermal extraction

**DOI:** 10.1002/fsn3.488

**Published:** 2017-06-02

**Authors:** Ololade Olatunji, Adetokunbo Denloye

**Affiliations:** ^1^ Department of Chemical Engineering University of Lagos Akoka Lagos Nigeria

**Keywords:** Biopolymer, collagen, fish, hydrolysis, kinetics, protein

## Abstract

This study investigates the kinetics of hydrolyzed collagen extraction from the scales of the croaker fish (*Pseudotolitus elongatus*) at temperature ranging from 60°C to 90°C. Extraction was carried out using hydrothermal treatment over a period of 8 hr, during which the mass of hydrolyzed collagen extracted was obtained every hour. The rate order of extraction was temperature‐dependent within the times investigated. At 60°C no measurable extraction was achieved, between 70°C and 80°C the extraction was a zero order while at 90°C and 100°C the extraction was a first order process. The rate constants for 70, 80, 90 and 100°C were 0.56 g s^−1^, 1.03 g s^−1^, 0.019 s^−1^ and 0.04 s^‐1^, respectively. The overall yield increased as temperature is increased with the highest increase in yield occurring between 90 and 100°C. The yield increased from 6% at 70°C to 30% at 100°C, thus indicating that temperature has significant effect on the yield as well as kinetics. These findings are relevant in the predictive assessment as well as design and optimization of processes for extraction of hydrolyzed collagen from fish scales.

## 1 INTRODUCTION

Fish scales are of research and economic significance due to the fact that they are a rich source of collagen and chitin which are two biopolymers of key importance in industries such as food and biomedical (Lin et al., 2010; Ikoma et al., 2003a,b)). Scales of fish is said to contain between 30 and 40% collagen by mass (Knorr, 1984; Torres, Troncoso, & Amaya, 2012; (Kumari & Rath, 2014). Collagen can be extracted from the scales through heat, acid, base or enzyme assisted hydrolysis or a combination of all or any of the aforementioned processes.

Thus far, research efforts have been directed at developing various means of extracting these biopolymers from fish scales as well as exploring various possible applications. Such studies include investigating the effect of various parameters such as temperature, pH, enzyme and time on the yield of hydrolyzed collagen from Fish scales (Zhang, Xu, & Wang, 2011), enzymatic extraction of acid soluble collagen (Wang et al., 2013a,b)among others. However, to the best of our knowledge there is yet to be a study which investigates the extraction kinetics of hydrolyzed collagen from fish scales using thermal extraction. Extraction time and temperature used in previous studies (Olatunji et al., 2014;Olatunji & Olsson, 2015) are based on parameters obtained from other studies which used alkali extraction (Wangtueai & Noomhorm, 2009; Zhang et al., 2011).

The process of extraction of proteins from scales of fish in the form of collagen or hydrolyzed collagen is affected by several parameters such as temperature, time, and species of the fish, pH and enzyme used. Although optimization studies have been carried out under enzymatic and acid based extraction using enzymes such as trypsin and pepsin (Wang et al., 2013a,b)., extraction of gelatine from fish scales using hydrothermal extraction is attractive as it is least complicated and least costly in terms of materials cost, given that it does not require use of enzymes or additional chemicals in the extraction process.

Previous studies which considered extraction of collagen from fish scales have mainly focused on the gel strength, microstructure, isotonic point and amino acid composition (Wang et al., 2013a,b; Zhang et al., 2011) using either acid, alkali or enzyme based extraction. For industrial process the product yield is also of huge significance.

To date there is no literature which provides data on the temperature and time‐dependent yield of hydrolyzed collagen from fish scale. Such data is necessary in process optimization and predictive assessment of the extraction process. The scales of croaker fish are of particular importance here as the croaker fish is one of the most abundant fish in the region of study (Nigeria) (Okoro, Aboaba, & Babajide, 2010).

The present study therefore aims at studying the extraction kinetics of hydrolyzed collagen from the scales of croaker fish (*Pseudotolitus* elongates) which is commonly found in the Nigerian marine waters (Okoro et al., 2010). The investigation will be limited to temperatures ranging between 60 and 100°C.

## 2 METHODOLOGY

### 2.1 Materials

Fish scales were obtained from a cold room in Abeokuta, Lagos. They were cleaned to get rid of debris and then dried in the sun till crisp dry. The scales were stored at room temperature in cardboard boxes and kept dry and aerated until needed. The sodium hydroxide and Hydrochloric acid used where reagent grade obtained from Sigma Aldrich (Supplied by Bristol Scientific, Lagos, Nigeria).

### 2.2 Method

#### 2.2.1 Pretreatment of fish scales

The initial stage was to demineralize the scales to get rid of the minerals (mainly calcium carbonate). To achieve this, 100 g of dried fish scales was treated with HCl at 4%w/v in the ratio 1:14 dry scales mass to volume of acid solution. This was kept in a beaker for 90 min at room temperature, swirling every 15 min. The scales were then washed with tap water repeatedly till neutral. The next stage involved removal of non collagen proteins through deproteinization with sodium hydroxide at a 1% w/v concentration in the ratio 1:3 wet scales to volume alkali solution. The scales are then repeatedly washed with tap water till neutral to get rid of the sodium hydroxide.

#### 2.2.2 Extraction of hydrolyzed collagen from Fish scales

The fish scales where strained to remove excess water and then transferred to a 4.5 L stainless steel pressure vessel, 300 ml of distilled water was added to the washed scales. The vessel was closed and heated to desired internal temperature. 40 ml samples are taken every hour for 8 hr and replaced with fresh distilled water in order to keep the volume constant. The extracted samples where centrifuged at 489 g for 15 min (Corning LSE, Compact Centrifuge, Sigma Aldrich, supplied by Bristol Scientific, Lagos, Nigeria) and the liquid separated from the solid residues. The liquid supernatant is then heated in a beaker at 80°C on a hot plate to evaporate water until it forms a thick viscous fluid. The viscous fluid was then spread on a flat polypropylene tops and allowed to dry in open air at room temperature (19 ± 5°C). The yield was obtained using Equation (1)

(1) $$\text{Yield}\;\;\%=\frac{\text{Mass of Hydrolysed Collagen}}{\text{Mass of Dry sclaes}}\times 100$$

To account for the 40 ml sample removed and replaced with distilled water, the concentration at given time is calculated using Equation (2).

(2) $$G_{n}^{*}=100-\left[\frac{\left[G_{n}+\left(\frac{40}{300}\times G_{n-1}\right)\right]}{40}\times 300\right]$$

where G_n_* is the mass of hydrolyzed collagen (g) remaining in the scales at any given time *t*_n_, G_n_ is the mass of hydrolyzed collagen in the 40 ml sample at time *t*_n_ and G_n‐1_ is the mass of hydrolyzed collagen in the sample at time *t*_n‐1_.

### 2.3 Protein analysis

The protein concentration for each extract was determined using Burette test and quantified using UV spectrometry at a wavelength of 540 nm. Standards were prepared using hydrolyzed collagen standard (Sigma Aldrich. Supplied by Bristol scientific Lagos, Nigeria).

### 2.4 Moisture content analysis

The moisture contents of the samples were obtained using the oven method. 0.5 g of scales were placed in an oven and dried at 180°C. The sample was taken out and weighed every 30 min until a constant weight is achieved. The moisture contents were calculated using Equation (3):

(3) $$\begin{array}{c} \text{Moisture content}\;\;\%=(\text{Initial mass before drying ‐ Final dry mass}\\ \times 100 \end{array}$$

### 2.5 Extraction rate measurement

The rate equation of each extraction is obtained from the mass of collagen released from the scales every hour over 8 hr. The mass of hydrolyzed collagen in the withdrawn sample is subtracted from the initial mass of the scales (100 g) and expressed as the residual mass of scales over time as shown in Equation (2). On completion of the 8 hr extraction, the process was stopped and bulk amount of hydrolyzed collagen extracted after 8 hr was measured in other to calculated total yield at the specified temperature as shown in Equation (1).

### 2.6 Statistical analysis

To quantify the level of significance of the values obtained *t* test was carried out on the results obtained in order to confirm if the patterns obtained are as a result of chance or reflect the reaction kinetics and the time and temperature‐dependent change in concentration.

## 3 RESULTS

The results presented here include the effect of temperature on the overall yield after 8 hours for all temperatures studied (60, 70, 80, 90, 100°C). We also present the reaction orders and rate constants obtained by plotting concentration of hydrolyzed collagen in scale against time. The results of protein concentration and moisture content are also presented.

### 3.1 Effect of temperature on the overall yield

**Figure 1:**
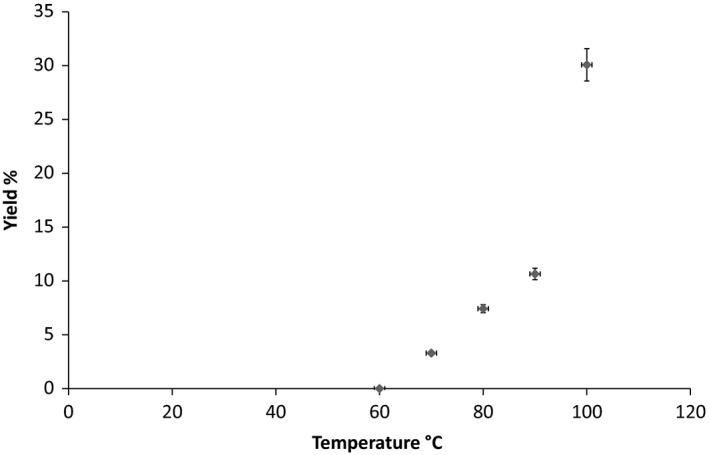
Yield at varying temperatures following 8 hr extraction time

**Figure 2:**
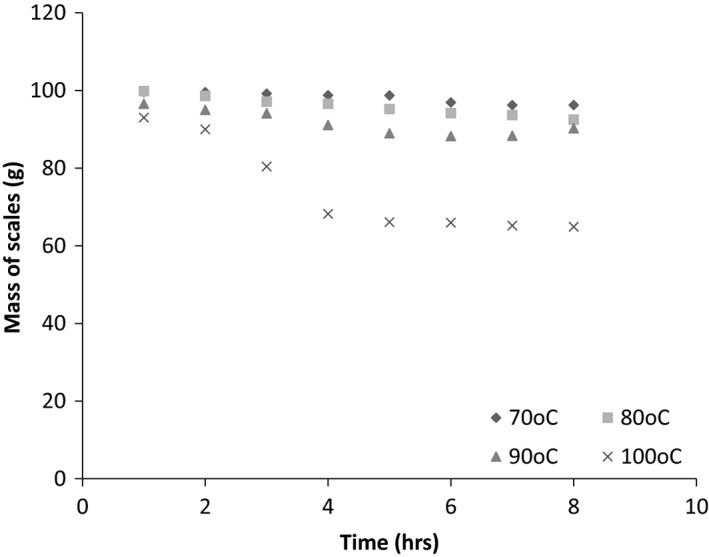
Mass of scales at varying extraction times

Figure 1 shows the yield plotted against temperature. Figure 2 shows the residual mass of scales at varying times. No extraction was obtained at 60°C and this is therefore not shown on the graphs. From the graphs it is observed that the mass of scales, hence the mass of hydrolyzed collagen extracted is temperature‐dependent and highest at 100°C.

### 3.2 Protein content

The protein content was measured using Burette test. The values are shown in Table 1 for all temperatures investigated. The results indicate that the protein concentration is unaffected by the temperature of extraction as no particular trend was observed. *p* > .2 indicated that the difference in values recorded was most likely due to chance thus indicating no dependency on temperature.

**Table 1:** Protein and moisture content of hydrolyzed collagen

Temperature (°C)	Protein content %	Moisture content %
70	87.7	11
80	88.7	11
90	88	11
100	88.5	11

### 3.3 Moisture content

In this study, precautions were taken to ensure that the moisture content was the same for all the samples by maintaining the same drying technique, drying and storage temperature as well as time. The samples were stored in sealed Ziploc bags which were doubled to avoid gaining or loosing moisture to the environment. To confirm this, moisture content analysis were carried out on the samples. It was observed that an average moisture content of 11% was maintained in the hydrolyzed collagen films.

### 3.4 Extraction kinetics of HC from fish scales

Table 2 summarizes the extraction kinetics data obtained from this study, the residual mass of scales is plotted against time to determine the rate order of the extraction process. To further determine the rate order, the graph of ln[M] and 1/[M] were also plotted. M represents the residual mass of the scales. The expression M is used as it relates to the mass of hydrolyzed collagen being removed from the scale under the assumption that the hydrolyzed collagen only is released into the water. This is a plausible assumption since it is based on previous studies; the hydrolyzed collagen is the only water‐soluble component of fish scales under the existing conditions (Ikoma et al., 2003a,b).

**Table 2:** Rate order and rate constant for extraction of hydrolyzed collagen from fish scales

Temperature	Rate order	Rate constant
70°C	Zero order	0.56 g s^−1^
80°C	Zero order	1.03 g s^−1^
90°C	First order	0.019 s^−1^
100°C	First order	0.04 s^−1^

At 60°C no measurable extraction was achieved therefore no result is presented for this temperature.

**Figure 3:**
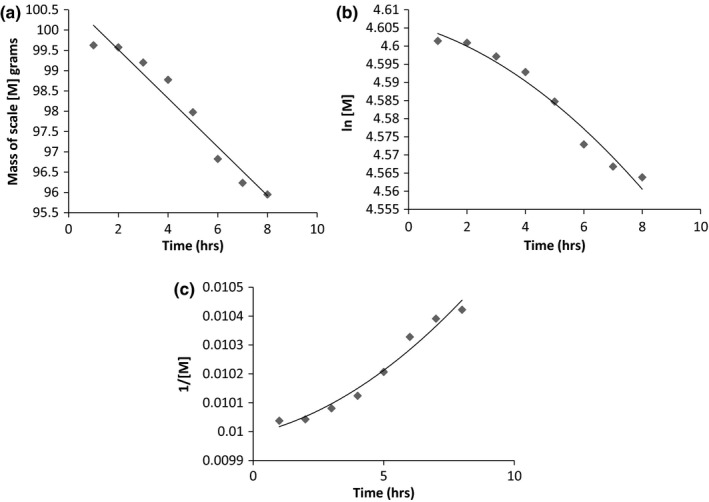
(a) Loss of mass of scales over time at 70°C. (b) Ln [M] against time at 70°C. (c) 1/[M] against time at 70°C

From Figure 3a–c, it is observed that extraction at 70°C is a zero order process. The process gives a linear graph when mass of scales is plotted against time. Using linear regression *R* ^*2*^ values of .95,.97 and .97, respectively were obtained for [M], ln[M] and 1/[M].

**Figure 4:**
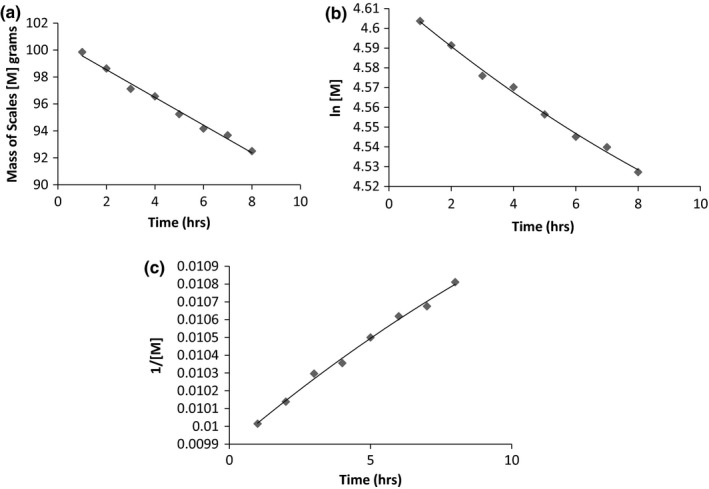
(a) Mass of scales against time at 80°C. (b) ln [M] versus time at 80°C. (c) 1/[M] versus time at 80°C

At 80°C, although a higher overall yield was obtained, the extraction maintained a zero order profile. Figure 4a–c shows the graph of mass of scales against time. The yield is almost doubled when the temperature increases from 70°C to 80°C such that although the process remains a zero order, it occurs at a faster rate. A reaction rate constant of 1.03 g s^‐1^ was obtained. Regression analysis was best fit at *R* ^*2* ^= .99 for all graphs indicating a good fit to the zero order kinetics. Statistical analysis indicates that the difference between the reaction order at 70°C and 80°C are significant with *p* = .0005.

**Figure 5:**
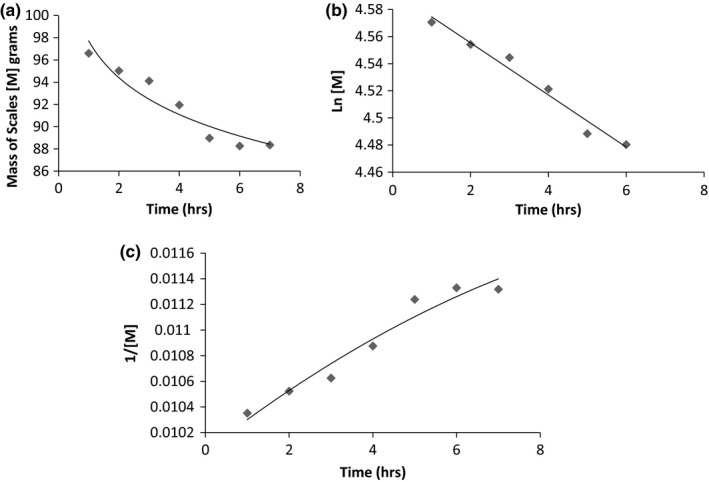
(a) Mass of scales versus time at 90°C. (b) Natural log of Mass of scales [M] plotted against time at 90°C. (c) Reciprocal of mass of scales plotted against time at 90°C

Increasing the temperature to 90°C resulted in the extraction changing from zero order to a first order process. Figure 5a–c shows the graph of concentration against time, the natural log of mass against time and the reciprocal of mass of scales against time. From linear regression the plots show good fit with *R* ^*2*^ values of .90, .97 and .95, respectively. The mass of hydrolyzed collagen extracted from the fish scales increased by a factor of 1.3 when temperature is increased from 80°C to 90°C. Statistical analysis indicates a *p* = .009 thus indicating that the difference in the values obtained are highly significant and not due to chance.

**Figure 6:**
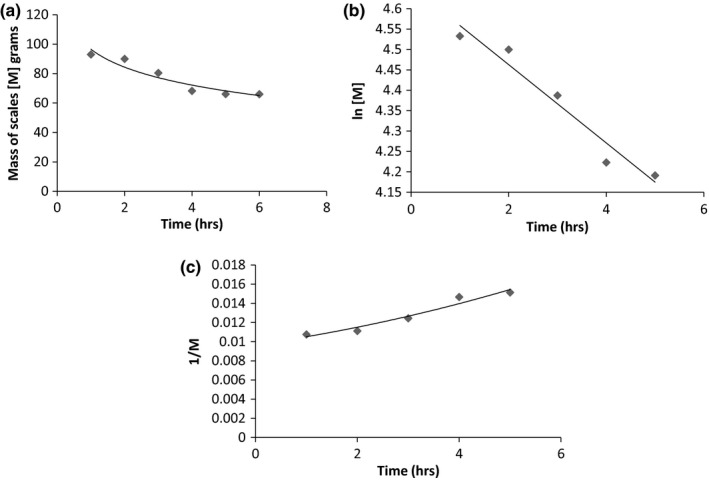
(a) Mass of scales at various times for 100°C extraction. (b) ln of mass of fish scales versus time at 100°C. (c) Plot of reciprocal of mass of scales over time at 100°C

At 100°C the maximum yield was obtained and the extraction process maintained a first order kinetics and a rate constant of 0.04 s^−1^. This is shown in Figure 6. For all repeated studies the yield increased significantly when the temperature is increased to 100°C with a *p* = .0038 indicating a low probability of the differences being due to just chance.

For this study the investigation was limited to 100°C. Beyond this temperature, evaporation occurred and this significantly distorted the result. The conclusions drawn from this study is therefore limited to the temperature ranged studied, 60–100°C.

## 4 DISCUSSION

The result indicates a positive correlation between yield and extraction temperature. The extraction temperature increased from no yield at 60°C to 30% yield at 100°C. Previous studies have recommended extraction of collagen using acid (Wang et al., 2013a,b) or enzymes (Zhang et al., 2011). Although using such methods collagen has been extracted from fish scales at optimal temperature of 30°C within 5.5 hr, here we look at an extraction process without the use of acid or enzymes which achieves a yield of up to 30% within 8 hr. Higher yields are also possible at prolonged hours (results not shown here) this will be further explored in our ongoing studies.

Our findings in this study indicate that for thermal extraction without the use of enzymes and acids, the yield can be improved by increasing the temperature. This therefore provides an alternative extraction process which is mainly heat‐ and time‐dependent.

The zero extraction obtained at 60 degrees is in agreement with previous reported studies in literature which recommend temperatures ranging between 70 and 80°C for extraction at lower pH values. This can be attributed to the fact that collagen is only soluble in water at higher temperature in the absence of enzymes or acids. A minimal temperature of 70°C is therefore required to rupture the orthogonal plywood structure of the fish scales by dissolving out the collagen hence breaking the triple helix structure.

## 5 CONCLUSION

The kinetics of extraction has been shown to be temperature‐dependent. The process is zero order at 70°C and becomes first order at 90°C. The yield of hydrolyzed collagen from fish scales of marine croaker is significantly increased by increasing temperature. Yield of up to 30% can be achieved using thermal extraction thus creating an alternative process to acid, alkali‐or enzyme‐based extraction of hydrolyzed collagen from fish scales. The results from this study are significant in the control and optimization of hydrolyzed collagen extraction process.

## CONFLICT OF INTEREST

Authors confirm no conflict of interest.
